# Morphological and molecular characterization of the parasite *Dipylidium caninum* infecting an infant in Colombia: a case report

**DOI:** 10.1186/s13071-022-05573-4

**Published:** 2022-12-13

**Authors:** Paula Benitez-Bolivar, Silvia Rondón, Mario Ortiz, Juana Díaz-Díaz, Cielo León, Juan Riveros, Helverth Molina, Camila González

**Affiliations:** 1 Centro de Investigaciones en Microbiología Y Parasitología Tropical (CIMPAT), Departamento de Ciencias Biológicas, Universidad de los Andes, Cra. 1 N° 18A-12, Bogotá, Colombia; 2 Unidad de Gastroenterología, Hepatología y Nutrición Pediátrica, Juan Pablo Riveros SAS, Bogotá, Colombia

**Keywords:** *Dipylidium caninum*, Dipylidiasis, Zoonosis, Colombia, 28S gene, human case

## Abstract

**Background:**

*Dipylidium caninum* is the causal agent of dipylidiasis affecting mainly cats and dogs worldwide. Human cases of dipylidiasis are rare, and the diagnosis is prevalently based on morphological features of the parasite. Here we report the diagnosis of dipylidiasis through morphological and molecular characterization of *D. caninum* infecting an 11-month-old boy in Cajicá, Colombia.

**Methods:**

Fresh faecal samples were obtained from the infant, and morphological identification of the parasite was performed through faecal smears. DNA was extracted from proglottids and used in PCR analyses for amplification of a 653-bp fragment of the nuclear ribosomal RNA (rRNA) encoding the 28S rRNA gene. A phylogeny study to better characterize the obtained DNA sequence was inferred using the maximum likelihood method and the Tamura-Nei model.

**Results:**

After morphological and molecular analyses, *D. caninum* was identified as the etiological agent causing the infection in the infant. Results of phylogenetical analyses showed that the obtained sequence clusters within the feline genotype clade. After the diagnosis of the parasite, effective treatment with praziquantel was administered to the infant.

**Conclusions:**

This is the third human case of dipylidiasis reported in Colombia, and the first study in South America to provide a molecular identification of *D. caninum.*

**Graphical abstract:**

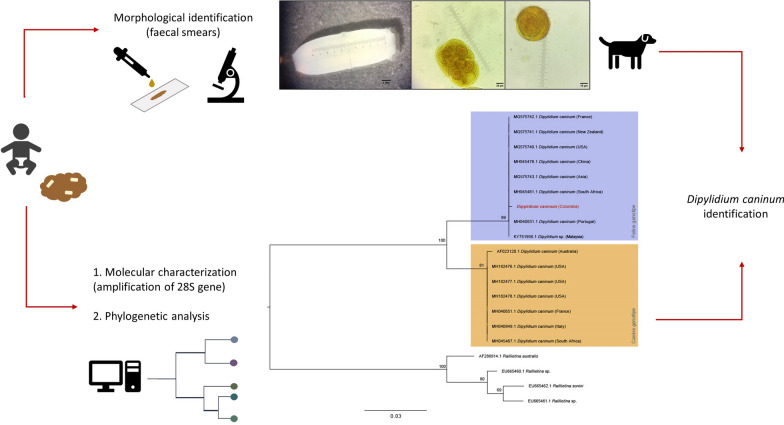

**Supplementary Information:**

The online version contains supplementary material available at 10.1186/s13071-022-05573-4.

## Background

Intestinal parasitic human infections caused by helminths or protozoans are among the most prevalent infections in developing countries, causing a high burden of morbidity and mortality. Specifically, economically disadvantaged children living in tropical and sub-tropical regions comprise the most affected population [1, 2]. Epidemiological evidence suggests that an estimated over one billion people in the world, mostly children, are infected with intestinal parasites [3]. The majority of these infections are due to *Ascaris lumbricoides*, *Trichuris trichiura* and hookworms, all with a global distribution and intensively transmitted in areas where more than 267 million preschool-age children and 568 million school-age children live [4]. The Pan American Health Organization (PAHO) estimates that the prevalence of helminths in Latin America and the Caribbean is greater than 20%, with the prevalence as high as 90% in low-resource areas [5]. According to the last national survey carried out between 2012 and 2014 in Colombia, 29.6% of the studied population was infected with some type of intestinal helminth [6], with the most prevalent parasite being *T. trichiura* (18.4%), followed by *A. lumbricoides* (11.3%) and hookworms (6.4%). Regarding cestodes, only five cases of *Taenia solium/saginata*, some cases of *Hymenolepis nana* (0.9%) and two cases of *Hymenolepis diminuta* were found [6]. However, a large number of cestode species have the ability to infect and colonize the intestinal tract of humans, as is the case of *Dipylidium caninum*.

*Dipylidium caninum* is a cestode that mainly infects cats and dogs, causing dipylidiasis. Dipylidiasis is a relatively common disease in these animals, but only incidently occurs in humans [7]. Definitive hosts become infected by feeding on intermediate hosts, such as fleas (*Ctenocephalides* spp. and *Pulex irritans*) and chewing lice (*Trichodectes canis* and *Felicola subrostratus*), that contain cysticercoids of the parasite [8]. Following the ingestion of these cysticercoids, the larval stage is released into the intestine of the definitive host, where it adheres by the scolex, and subsequently the adult parasite develops. Gravid proglottids are shed in the host’s faeces and then fragment into the environment, releasing the eggs and thus allowing the cycle to continue.

Although numerous dipylidiasis cases have been reported in humans, most clinicians still consider this parasitosis as unusual [9]. Children have the greatest risk of infection by accidental ingestion of fleas due to hand-mouth contact after touching and caressing pets [10]. In fact, most reports of human dipylidiasis cases have been registered in infants, with a worldwide distribution: Ethiopia, India, Spain, Poland, USA, Russia, Costa Rica, Venezuela and Chile, among others [11–19]. The presence of *D. caninum* eggs in pre-Columbian human coprolites found in Puerto Rico and dated from 180 A.D. to 600 A.D. has also been reported [20].

In Colombia, there are two records of dipylidiasis in humans, one from a child in the northern coastal region [21] and one from an adult woman successfully treated with praziquantel [22]. Also in Colombia, *D. caninum* infection has been reported in cats and dogs in Medellin [23], in dogs in Bogota [24] and Tolima Department [25] and in hunting dogs belonging to indigenous communities in Vaupes Department [26]. Additionally, a study of samples from dogs and children in Valle del Cauca Department reported *D. caninum* infections only in dogs [27].

Treatment of patients infected with cestodes is key to eliminating the parasites and improving the patient’s health status. In this context, the parasite must be correctly identified in order to provide the patient with the appropriate and most effective treatment. A general lack of clinical and diagnostic information on these uncommon cestode infections often results in heathcare providers confusing *Dipylidium* infection with pinworm infection, primarily based on the symptoms of itching in the anal area and the movement of the gravid proglottids in the faeces [9]. To achieve an appropriate morphological identification, characteristics of the parasite, such as the presence of scolex, proglottids, shape and size of the eggs, among other factors, must be considered [9]. Molecular biology techniques also provide a rapid and reliable way to improve the detection and epidemiological tracking of dipylidiasis in humans and in other animals [28].

Here, we report a case of dipylidiasis in an infant in Colombia that was identified through molecular and morphological identification of the parasite, with a phylogenetic analysis based on the 28S ribosomal RNA (rRNA) gene.

## Methods

### Case report

The parents of an 11-month-old patient residing in the municipality of Cajicá, Cundinamarca Department, Colombia consulted the paediatric service in March 2020 due to a 15-day evolution of expulsion of white mobile forms in the stool of the infant. The initial treatment provided by the paediatric service was the administration of pyrantel pamoate, an antiworm medication. As no clinical improvement was noted, a subsequent treatment with albendazole was administered, also with no effect. Therefore, metronidazole was administered for 7 days. The family was referred to the paediatric gastroenterology service in May 2020. The parents provided a video of a diaper with evident mobile structures (Additional file 1: Video S1). The parents denied other associated symptoms. When asked about their daily activities and domestic situation, the parents reported a pet (dog) and that they regularly took the child to a green area in the zone where they live. The child frequently crawled on the grass. Physical examination was completely normal, with adequate weight and height gain. No signs of micronutrient deficiencies were evident. Additional medical studies were carried out, ruling out iron deficiency anaemia and confirming normal liver function and normal primary immunodeficiency. Two fresh faecal samples were collected from the patient and sent to the Centre for Research in Microbiology and Tropical Parasitology (CIMPAT) at Universidad de Los Andes (Bogotá, Colombia) for analysis.

### Morphological analyses

Faecal samples were macroscopically examined, and faecal smears were subsequently performed using microscope slides with 1% iodine solution and 0.85% saline solution [29]. Slides were examined with a microscope, using the objectives 10×, 40× and 100×. Measurements of capsules, eggs and proglottids were determined using the ImageJ software.

### Molecular analysis

DNA from proglottids was extracted using a High Pure PCR Template Preparation Kit (Roche Life Sciences, Penzberg, Germany), according to the manufacturer’s protocol. PCR was conducted using the *D. caninum*- specific reverse primer DFC28S-1R (5′-CACATTCAACGCCCGACTCCTGTAG-3′) and the forward primer DC28S-1F (5′-GCATGCAAGTCAAAGGGTCCTACG-3′) for amplification of a 653-bp fragment of the nuclear rRNA encoding the 28S rRNA gene [30, 31]. PCR products were visualized on an agarose gel, and Sanger sequencing was subsequently performed. The obtained sequence was edited using the CLC Genomics Workbench Software. The reverse complement was done with GeneRunner and compared with publicly available sequences using BLAST (National Center for Biotechnology Information, Bethesda, MD, USA).

### Phylogenetic analysis

After identifying the species through BLAST, a phylogenetic analysis was performed to better characterize the obtained sequence. To build the tree, we downloaded from GenBank a total of 15 sequences of the 28S rRNA gene from specimens belonging to *D. caninum* (Table 1). The sequences were representative of both the canine and feline genotypes that are characterized within the species [32] and were taken from specimens found in different locations across all continents. Additionally, four sequences of the same gene belonging to the genus *Raillietina* were downloaded and used as the outgroup. Sequences were aligned using MUSCLE, and the phylogeny was inferred using the maximum likelihood method and the Tamura-Nei model [33]. All positions with < 90% site coverage were eliminated; i.e. positions with < 10% alignment gaps, missing data and ambiguous bases were allowed at any position (partial deletion). Finally, the final dataset contained a total of 419 positions and 20 sequences. Both the alignment and the phylogenetic analysis were performed using the program MEGA X [34].

**Table 1:** Information on the sequences obtained from GenBank and used in the phylogenetic analysis

GenBank accession number	Species	Genotype	Geographic origin	Length (bp)	References
ON509896	*Dipylidium caninum*	Feline	Colombia	564	Sequence generated in this study
MG575742.1	*Dipylidium caninum*	Feline	France	649	[35]
MG575741.1	*Dipylidium caninum*	Feline	New Zealand	640	[35]
MG575740.1	*Dipylidium caninum*	Feline	USA	651	[35]
MH045476.1	*Dipylidium caninum*	Feline	China	604	[35]
MG575743.1	*Dipylidium caninum*	Feline	Asia	645	[35]
MH045481.1	*Dipylidium caninum*	Feline	South Africa	604	[35]
MH040831.1	*Dipylidium caninum*	Feline	Portugal	604	[35]
KY751956.1	*Dipylidium caninum*	Feline	Malaysia	569	[36]
AF023120.1	*Dipylidium caninum*	Canine	Australia	608	[37]
MH182476.1	*Dipylidium caninum*	Canine	USA	632	[31]
MH182477.1	*Dipylidium caninum*	Canine	USA	632	[31]
MH182478.1	*Dipylidium caninum*	Canine	USA	653	[31]
MH040851.1	*Dipylidium caninum*	Canine	France	606	[35]
MH040849.1	*Dipylidium caninum*	Canine	Italy	606	[35]
MH045467.1	*Dipylidium caninum*	Canine	South Africa	606	[35]
AF286914.1	*Raillietina australis*		Australia	590	[38]
EU665460.1	*Raillietina sp.*		USA	423	[39]
EU665462.1	*Raillietina sonini*		Bulgaria	424	[39]
EU665461.1	*Raillietina sp.*		USA	424	[39]

## Results

### Morphological analyses

**Fig. 1:**
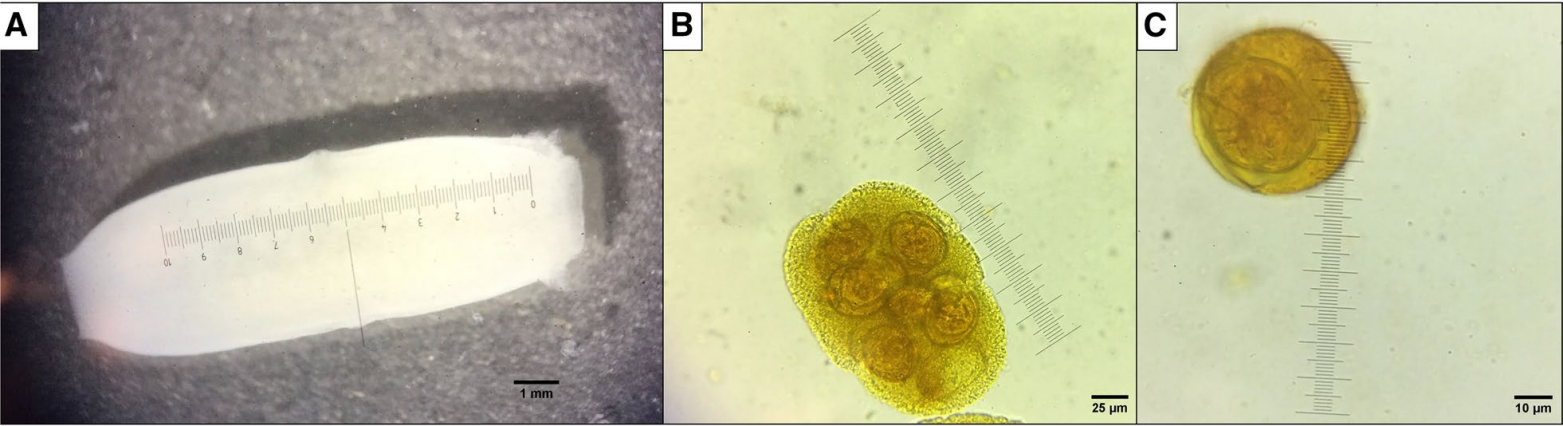
**A** Double-pored proglottid, **B** egg package (40× magnification, lugol solution staining), **C** egg with visible oncosphere and hexacanth (100× magnification, lugol solution staining)

Macroscopically, parasitic forms with white colouration, measured to be 14 mm in length and 4.8 mm in width (measurement taken from pore to pore), each with the presence of two equatorial genital pores, were observed. These parasitic forms correspond to proglottids, with characteristics of the Cestoda class (Fig. 1a). Microscopically, using the objectives 10× and 40×, capsules with eggs were observed and measured (160 µm in length and 120 µm in width with the 40× objective) (Fig. 1b). Eggs were also observed in detail using the 100× objective, with each egg showing a roughly spherical shape (diameter: 44 µm) and an inner oncosphere with hooks (Fig. 1C).

### Molecular analysis

The aligned sequenced product size was 564 bp. The amplicon was identified as *D. caninum* based on the best match in BLAST showing 100% of identity (MH045481.1). The sequence MH045481 had been isolated from a worm found in a cat host in the South Africa region [32].

### Phylogenetic analysis

**Fig. 2:**
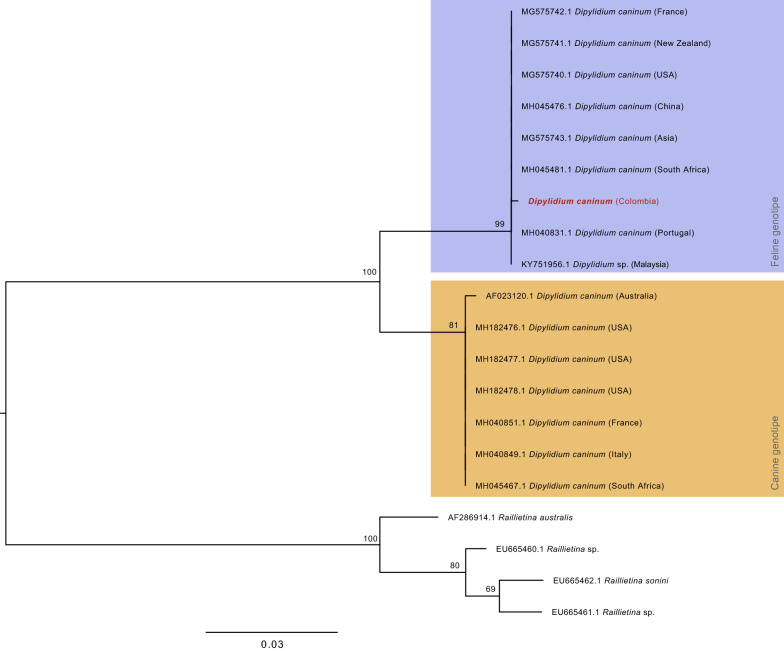
Phylogenetic tree with the highest log likelihood (- 1067.18), obtained from phylogenetic analysis of 28S sequences of *Dipylidium caninum* and *Raillietina* sp. (Table 1). The tree was built using the maximum likelihood method and the Tamura-Nei model [33]. The phylogeny is drawn to scale, with branch lengths measured in the number of substitutions per site; bootstrap values are shown above each branch. Each specimen is identified by its GenBank accession number, species name and the country where it was collected. The sequence obtained from the Colombian specimen of this study is highlighted in red. Additionally, the two clades formed by the feline and canine genotypes are shown in purple and yellow, respectively

The phylogeny produced using the maximum likelihood method and 16 partial 28S rRNA gene sequences from *D. caninum* specimens, including the newly generated sequence from Colombia, is shown in Fig. 2. The two genotypes described by Labuschagne et al. [32], namely a canine genotype and feline genotype, form two distinct clades regardless of the geographical origin of the specimens. Interestingly, the Colombian sequence clusters within the feline genotype clade support that the specimen has the genotype related to the cat host.

## Discussion

To the best of our knowledge, this is the third human case of dipylidiasis reported in Colombia, and the first report in South America of a molecular analysis of the sample retrieved.

The two previous human cases of *D. caninum* infection reported in Colombia [21, 22], as well as the infection in cats and dogs [23–27], were diagnosed based on the morphology of the parasite. In the present study, morphological and molecular approaches were used as complementary diagnostic tools. Molecular tools were used not only to identify the parasite causing the infection but also to study the phylogenetic relationships of a *D. caninum* DNA sequence obtained from a human host*,* especially its relationship with the previously proposed feline and canine genotypes [32].

After the identification of the parasite, the infant received effective antiparasitic treatment with praziquantel at a dose of 25/mg/kg/day divided into three doses for 2 days. Additionally, as the infant’s family reported having a flea-infested dog at home, the dog was also treated with praziquantel. Fortunately, in both the infant and the dog the treatment was successful, as clinical resistance to praziquantel in *D. caninum* has been previously reported. Future epidemiological studies to elucidate the mechanism of resistance are encouraged [31]. Parasitic treatment of pets, flea control, proper disposal of pet’s faeces, hand hygiene after caressing pets and discouraging children to play in areas soiled with pet’s faeces are some of the factors preventing human dipylidiasis [15].

The 28S rRNA gene harbors different regions that evolve at varying rates and therefore has been used to estimate relationships within and among the Platyhelminthes; in fact, it is one of the most commonly targeted genes used to identify cestodes [28]. In the present study, the phylogenetic analysis using the 28S rRNA gene showed a clustering of the obtained sequence within the feline genotype clade of *Dipylidium* that includes sequences from different countries (i.e. France, New Zealand, South Africa, Malaysia). As a pet dog infested with fleas was probably the source of infection of the infant, the phylogenetic analysis was expected to obtain a clustering of the retrieved sequence into the canine lineage. However, the authors of a previous study found: (i) that 2–10% of *D. caninum* DNA extracted from cats or *Ctenocephalides felis* collected from cats belong to the canine genotype; and (ii) similarly, *D. caninum* DNA extracted from dogs or *C. felis* collected from dogs belong to the feline genotype [35]. Additionally, Beugnet et al. [35] found out that dogs could be experimentally infected by *Dipylidium*'s feline lineage.

## Conclusions

A case of dipylidiasis in an infant in Colombia is reported. Morphological and molecular identification of the parasite was performed, thus also contributing to the knowledge of the molecular epidemiology of human dipylidiasis in South America. Although *D. caninum* is rarely found infecting humans, the importance of the molecular identification of this parasite and the reporting of human cases is highlighted to adequately determine its significance in public health.

## Supplementary Information

**Additional file 1: Video S1.** Infant stool with moving *Dipylidium caninum* proglottids.

## Data Availability

All data generated or analysed during this study are included in this published article and its supplementary file. Our sequence was submitted to the NCBI platform under GenBank accession number ON509896.
